# A yeast‐based tool for screening mammalian diacylglycerol acyltransferase inhibitors

**DOI:** 10.1002/mbo3.1334

**Published:** 2022-12-01

**Authors:** Peter Gajdoš, Rodrigo Ledesma‐Amaro, Jean‐Marc Nicaud, Tristan Rossignol

**Affiliations:** ^1^ Institute of Biotechnology, Faculty of Chemical and Food Technology Slovak University of Technology Radlinskeho Bratislava Slovakia; ^2^ Department of Bioengineering, Imperial College Centre for Synthetic Biology Imperial College London, South Kensington Campus London UK; ^3^ Université Paris‐Saclay, INRAE, AgroParisTech, Micalis Institute Jouy‐en‐Josas France

**Keywords:** acyl‐CoA:diacylglycerol acyltransferase, DGAT, heterologous expression, inhibitor screening, *Yarrowia lipolytica*

## Abstract

Dysregulation of lipid metabolism is associated with obesity and metabolic diseases but there is also increasing evidence of a relationship between lipid body excess and cancer. Lipid body synthesis requires diacylglycerol acyltransferases (DGATs) which catalyze the last step of triacylglycerol synthesis from diacylglycerol and acyl‐coenzyme A. The DGATs and in particular DGAT2, are therefore considered potential therapeutic targets for the control of these pathologies. Here, the murine and the human DGAT2 were overexpressed in the oleaginous yeast *Yarrowia lipolytica* deleted for all DGAT activities, to evaluate the functionality of the enzymes in this heterologous host and DGAT activity inhibitors. This work provides evidence that mammalian DGATs expressed in *Y. lipolytica* are a useful tool for screening chemical libraries to identify potential inhibitors or activators of these enzymes of therapeutic interest.

## Graphical abstract

The diacylglycerol acyltransferase (DGAT) enzymes catalyze the final step of the triacylglycerol biosynthesis by esterification of a fatty acyl moiety to a diacylglycerol. Murine and human DGAT2 were overexpressed in the strains of oleaginous yeast *Yarrowia lipolytica* deleted for all DGAT activities. Heterologous DGAT functionality was validated and response specificity to known DGAT inhibitors was evaluated. This work provides proof of principle for using these strains as a screening system for libraries of molecules to discover new inhibitors or activators of these enzymes of particular therapeutic interest.

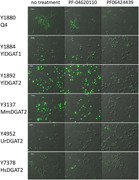

## 1 INTRODUCTION

The diacylglycerol acyltransferase (DGAT) enzymes catalyze the final committed step of the triacylglycerol (TAG) biosynthesis by esterification of a fatty acyl moiety to a diacylglycerol. These neutral lipids are stored in organelles called lipid bodies (LBs) in mammalian adipose tissue but also in most eukaryotic cells and some prokaryotes as energy molecules or membrane synthesis reservoirs. In eukaryotes, TAGs are mainly synthesized by DGAT1 and DGAT2 (two different gene families). DGAT1 and DGAT2 have different roles in TAG synthesis in humans: DGAT1 is highly expressed in the small intestine and has a role in fat absorption while DGAT2 is expressed in liver and adipose tissue and is responsible for the endogenous synthesis of TAG (Cases et al., 1998, 2001). The *dgat1* knockout mice are viable with a minor impact on TAG levels and are resistant to diet‐induced obesity (Smith et al., 2000). In contrast, *dgat2* knockout mice present severe TAG decrease and die shortly after birth (Stone et al., 2004). And, TAG excess in tissues is a hallmark of obesity. Therefore, DGATs are considered potential therapeutic inhibition targets for the control of obesity, but also for some diseases related to lipid absorption in the intestine. Moreover, recent studies revealed that high levels of LBs are also associated with breast cancer (Nisticò et al., 2021) as well as with higher tumor aggressiveness and chemotherapy resistance (Tirinato et al., 2017). Interestingly DGAT2 is constitutively activated in various cancers including breast cancer (Hernández‐Corbacho & Obeid, 2019). In addition, the importance of DGAT2‐mediated regulation of TAG metabolism in triple‐negative breast cancer has been recently highlighted (Almanza et al., 2022). Therefore, DGAT2 appears as a new potential therapeutic target in the treatment of breast cancer (Hernández‐Corbacho & Obeid, 2019). Because of the lethality of *dgat2* knockout mice model, specific inhibitors that tightly control the inhibition of DGAT2 are required. Compound libraries targeting obesity as well as cancer should be evaluated in a system that could mimic human and mouse DGAT2 structure and activity with ease and high throughput screening capacity.

Being able to express these enzymes in a simple heterologous model would provide an efficient and versatile tool to characterize these enzymes and potential inhibitors. To do so, in this work, the oleaginous yeast *Yarrowia lipolytica* has been used as a heterologous host. This yeast is particularly valuable in this context. It has been a model for lipid metabolism for decades and has a high enzyme production capacity (Nicaud, 2012). Additionally, it can produce large LBs and it is easy to manipulate thanks to the numerous modern genetic engineering toolboxes now available (Larroude et al., 2018). In particular, a strain deleted for all the genes coding for enzymes with DGAT activities (Q4) is available (Beopoulos et al., 2012). This strain is not able to form LBs anymore. Previous work has shown that DGAT activity in *Y. lipolytica* can be easily validated, characterized, and modulated by overexpression approaches in this genetic background, allowing restoration of LB formation and TAG accumulation (Aymé et al., 2015; Gajdoš et al., 2016, 2019). Those previous works established the efficiency and versatility of the heterologous expression of DGAT in this particular host.

To determine whether the heterologous constructs could potentially be used as tools to measure the activity of these enzymes and thus useful for screening chemical libraries to identify regulatory molecules, the murine and the human DGAT2 were overexpressed in the above‐mentioned Q4 strain, as well as the oleaginous fungus *Umbelopsis rhamaniana* DGAT2, the first DGAT2 identified and expressed in a heterologous host (Lardizabal et al., 2001), and the *Y. lipolytica* DGATs for comparison. MmDGAT2 and HsDGAT2 have already been expressed in heterologous systems including insect cells and yeast, but mainly for in vitro activity assays (Cases et al., 2001; Kim et al., 2014; Stone et al., 2006; Turkish et al., 2005; Yen et al., 2005). The DGAT overexpression in the Q4 chassis strains was therefore first evaluated for the LB restoration phenotype and TAG accumulation to evaluate the capacity to use them as in vivo screening tools for DGAT inhibitor candidate drugs. These strains were then exposed to known inhibitory molecules of mammalian DGAT1 and DGAT2. The results showed that the DGATs are functional in our chassis and that inhibitors conserved their specificities and efficacy. This work provided proof of principle for using these strains as a screening system for libraries of molecules to discover new inhibitors or activators of these enzymes of particular therapeutic interest.

## 2 MATERIAL AND METHODS

### 2.1 Compounds, media, and culture conditions

Both PF‐06424439 and PF‐046020110 were obtained from Sigma‐Aldrich. Stock solutions were prepared by resuspending the powder at 5 mg/mL in sterile distilled water for PF‐06424439 and in pure DMSO for PF‐046020110. The *E. coli* strains were grown in lysogeny broth medium complemented with 50 μg/mL kanamycin or 100 μg/mL ampicillin when required. For yeast growth and transformant selection, minimal YNB medium, composed of 0.17% (w/v) yeast nitrogen base (without amino acids and ammonium sulfate), 0.5% (w/v) NH_4_Cl, 50 mM phosphate buffer (pH 6.8), and 2% (w/v) glucose was used. Leucine was added at a final concentration of 0.1 g/L when required. For higher lipid accumulation experiments, similar YNB medium with 0.15% (w/v) NH_4_Cl and 3% (w/v) glucose was used, which corresponded to a carbon‐to‐nitrogen ratio of 30 (C/N 30). Solid media were complemented with 1.6% agar.

For growth in 96‐well microtiter plates, yeasts were precultured in YNB overnight at 28°C, washed, and diluted in fresh YNB medium at an optical density (OD) at 600 nm of 0.2. 100 µL of this dilution was mixed with 100 µL of inhibitor solution diluted in YNB at the required concentration. Cultures were grown at 28°C under constant agitation on a Biotek Synergy MX microtiter plate reader (Biotek Instruments) and monitored by measuring OD at 600 nm every 20 min for 72 h. Growth rates (*r*) were calculated using the Growthcurver R package (Sprouffske & Wagner, 2016) on 6 biological replicates.

For flask culture, yeasts were pre‐cultured in YNB overnight at 28°C, washed and diluted at an OD at 600 nm of 0.2 in 10 mL of fresh YNB C/N 30 containing inhibitors at the indicated concentration and grown at 28°C under constant agitation at 160 rpm. For lipid extraction and quantification, cells were grown in triplicates.

### 2.2 Plasmid and strains construction

*UrDGAT2* (GenBank accession number: AAK84179.1) was synthesized and codon optimized by Genscript. The gene was cloned under the pTEF promoter between BamHI and AvrII cloning sites in the overexpression JMP62 vector (Nicaud et al., 2002) containing the selective *URA3* marker*,* to generate JMP2881 plasmid. *MmDGAT2* were PCR‐amplified from cDNA cloned vectors (Yen et al., 2005) using primers that allowed Gateway cloning by introducing attb sequences (Attb1‐DGAT2_forward GGGGACAAGTTTGTACAAAAAAGCAGGCTATGAAGACCCTCATCGCCGCCTACTCCGGG; Attb2‐DGAT2_reverse GGGGACCACTTTGTACAAGAAAGCTGGGTCTCAGTTCACCTCCAGCACCTCAGTCTCTG). The PCR fragments were cloned into the Gateway® vector pDONR207 (Invitrogen) using Gateway BP clonase (Thermo Fisher Scientific) to generate plasmid JMP1783 and transferred into the *Y. lipolytica* Gateway expression vector JMP1529 (Leplat et al., 2015) using Gateway LR clonase (Thermo Fisher Scientific) giving rise to the plasmids JMP1785 (*pTEF‐MmDGAT2‐URA3ex*). The *HsDGAT2* cDNA clone (NM_032564) was bought from Genscript and was amplified with the same primers as for *MmDGAT2* (one nucleotide difference and no change in the amino acid sequences) to remove the C‐terminal tag present in the vector and introducing the attb sequences for Gateway® cloning. PCR fragments were cloned into the Gateway® vector pDONR207 (Invitrogen) to generate plasmid JME4451 and transferred into the *Y. lipolytica* Gateway expression vector JMP1529 (Leplat et al., 2015), giving rise to the plasmids JMP4468 (*pTEF‐HsDGAT2‐URA3ex*). The NucleoSpin Plasmid EasyPure kit (Macherey‐Nagel) was used for plasmid purification and expression cassettes were sequence verified. Expression cassettes from the *NotI*‐digested plasmids JMP2881, JMP1785, and JMP4468 were used for *Y. lipolytica* transformation in the Y1877 strain (the Q4 strain) which lacked the four acyltransferases (Beopoulos et al., 2012) using the lithium acetate method (Le Dall et al., 1994), creating strains Y4952 (Q4‐UrDGAT2), Y3137 (Q4‐MmDGAT2) and Y7378 (Q4‐HsDGAT2), respectively. Strains Y1880 (Q4), strains Y1884 (Q4‐YlDGAT1) and Y1892 (Q4‐YlDGAT2) were described previously (Beopoulos et al., 2012). All the plasmids and strains used in this study are listed in Tables 1 and 2, respectively.

**Table 1:** List of plasmids used in this study

Plasmids	Genotype	References
JMP1529	Gateway expression vector	Leplat et al. (2015)
JMP1046	Expression vector	Nicaud et al. (2002)
JMP2881	JME1046 + UrDGAT2	This work
JMP1783	pDONR207 + MmDGAT2	This work
JMP1785	JMP1529 + MmDGAT2	This work
JMP4451	pDONR207‐HsDGAT2	This work
JMP4468	JME1529‐HsDGAT2	This work

**Table 2:** List of *Yarrowia lipolytica* strains used in this study

Strains	Genotype	References
Y1877 (Q4)	*leu2‐270 ura3‐302 Δdga1Δlro1Δare1Δdga2*	Beopoulos et al. (2012)
Y1880	*leu2‐270 ura3‐302 Δdga1Δlro1Δare1::URA3 Δdga2*	Beopoulos et al. (2012)
Y1884	*leu2‐270 ura3‐302 Δdga1Δlro1Δare1Δdga2 pTEF‐YlDGAT1‐URA3ex*	Beopoulos et al. (2012)
Y1892	*leu2‐270 ura3‐302 Δdga1Δlro1Δare1Δdga2 pTEF‐YlDGAT2‐URA3ex*	Beopoulos et al. (2012)
Y3137	*leu2‐270 ura3‐302 Δdga1Δlro1Δare1Δdga2 pTEF‐MmDGAT2‐URA3ex*	This work
Y4952	*leu2‐270 ura3‐302 Δdga1Δlro1Δare1Δdga2 pTEF‐UrDGAT2‐URA3ex*	This work
Y7378	*leu2‐270 ura3‐302 Δdga1Δlro1Δare1Δdga2 pTEF‐HsDGAT2‐URA3ex*	This work

### 2.3 Fluorescence microscopy

For LB staining, cells were stained at room temperature by a 10‐min incubation with BODIPY 493/503 (Invitrogen) at 1 μg/mL. Images of living stained cells were taken using a Zeiss Axio Imager M2 microscope equipped with an HXP 120 C lamp (Zeiss), with a 100X oil immersion objective, a Zeiss fluorescence microscopy filter set 45 for the detection of BODIPY and phase contrast. AxioVision 4.8 software (Zeiss) was used for observing and recording images using an exposure time of 300 ms for filter 45 (BODIPY). Cultures for staining and imaging were done at least in duplicates for confirmation of the visual phenotype.

### 2.4 Fluorescence quantification

For fluorescence quantification, cells were grown in 96 well plates as described in (Morin et al., 2014) culture conditions section. BODIPY and potassium iodide were added to evaluate BODIPY fluorescence relative to OD. Fluorescence and OD were measured after 60 h of growth. For strain evaluation against the different inhibitors, we used two biological replicates. Average and standard deviation values as well as *p*‐values were calculated using a Welch *t*‐test.

### 2.5 Sequence analysis

Sequence alignments and the neighbor‐joining phylogenetic tree were performed using Clustal Omega (https://www.ebi.ac.uk/Tools/msa/clustalo/). Protein domain identifications were performed using Interpro (https://www.ebi.ac.uk/interpro/).

### 2.6 Lipid extraction and quantification

For dry cell weight (DCW) determination, the culture was washed two times with distilled water and lyophilized in a preweighed tube. The differences in mass corresponded to the mg of cells found in the culture. Lipids were extracted from 10 to 20 mg of dried cells and converted into FA methyl esters (FAMEs) using the procedure described by Browse et al. (1986) with cyclohexane instead of hexane. Briefly, dried biomass (10–20 mg) was mixed with 1 mL of 2.5% (v/v) sulfuric acid in methanol, which contained 25 μg of commercial dodecanoic acid (Sigma‐Aldrich) as an internal standard. Tubes were vortexed and incubated at 80°C for 120 min to form FAMEs. After transesterification, 1 mL of cyclohexane and 0.5 mL of distilled water were added. The FAME‐containing cyclohexane phase was analyzed by gas chromatography (GC) using a Varian 430 instrument (Varian Inc.) equipped with a flame ionization detector and a Varian FactorFour vf‐23 ms column, where the bleed specification at 260°C was 3 pA (30 m, 0.25 mm, 0.25 μm). The FAMEs were identified by comparison with commercial standards (FAME32; Supelco) and quantified using dodecanoic acid as an internal standard. For each condition, we used three biological replicates and calculated average and standard deviation values. The *p*‐values were calculated using a Welch *t*‐test.

## 3 RESULTS AND DISCUSSION

### 3.1 LB forming phenotype complementation

**Figure 1:**
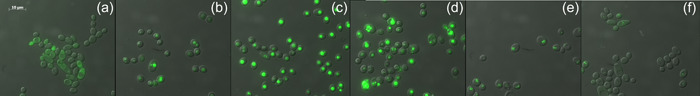
Phenotype complementation of strains overexpressing diacylglycerol acyltransferases (DGAT). (a) Strain Y1880 Q4. (b) Strain Y1884 Q4 + YlDGAT1. (c) Strain Y1892 Q4 + YlDGAT2. (d) Strain Y3137 Q4 + MmDGAT2. (e) Strain Y4592 Q4 + UrDGAT2. (f) Strain Y7378 Q4 + HsDGAT2.

**Figure A1:**
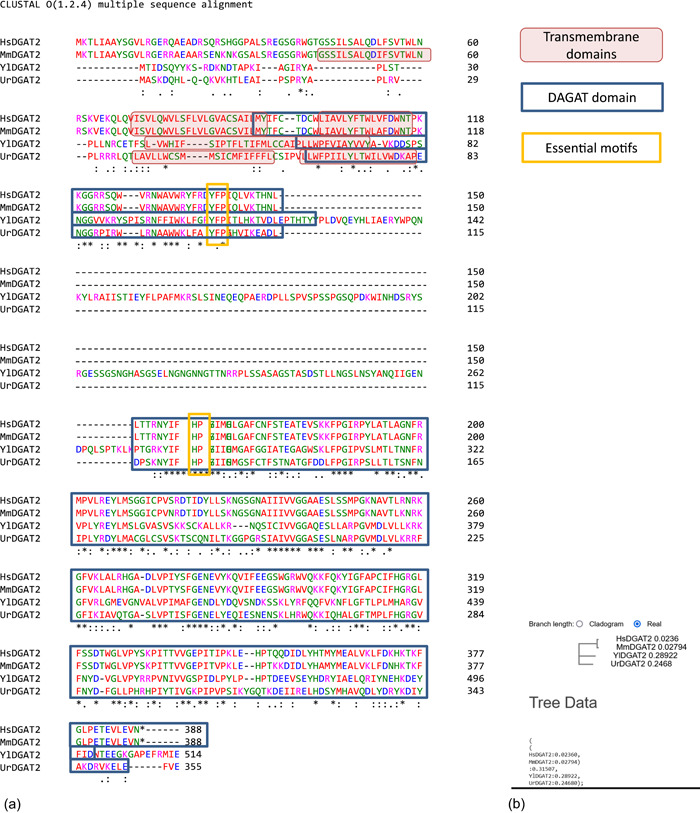
(a) Protein sequence alignment. (b) Phylogenetic tree.

The Q4 chassis strain used in this study is unable to form LBs. Therefore, heterologous DGAT functionality can be easily evaluated by simple observation of LB restoration directly supporting enzymatic activity in the heterologous host. The *Y. lipolytica* YlDGAT1 and YlDGAT2 overexpressed in the Q4 chassis were previously validated (Beopoulos et al., 2012; Gajdoš et al., 2016) and serve as controls for the Q4 strains overexpressing MmDGAT2, UrDGAT2, and HsDGAT2. All strains show a complementation phenotype by the formation of LBs (Figure 1). Thus, YlDGAT2 overexpression is the highest as expected, as it is the main DGAT for lipid accumulation in LBs in *Y. lipolytica* (Gajdoš et al., 2016), while that of HsDGAT2 is the lowest in the condition tested with small LB formation. Protein sequence alignment shows that all the DGAT2 tested here share the essential YFP and HPHG motifs (Liu et al., 2012), however, there is a very large stretch between these two motifs in YlDGAT2 compared to the other DGAT2 (Figure A1a). Despite this important structural difference and the fact that the codons usage of the heterologous MmDGAT2 and HsDGAT2 gene were not optimized for expression in *Y. lipolytica*, all the heterologous DGAT2 are functional in *Y. lipolytica*. MmDGAT2 performed nearly as well as the yeast DGAT2 did in the mutant *Y. lipolytica* strains. It seems that the long hydrophilic sequence between the two important conserved motifs had relatively little effect on DGAT activity. While MmDGAT2 and HsDGAT2 have a strong homology (Figure A1b), HsDGAT2 has a lower activity in this particular host.

### 3.2 Evaluation of DGAT2 inhibitor activity on HsDGAT2

**Figure 2:**
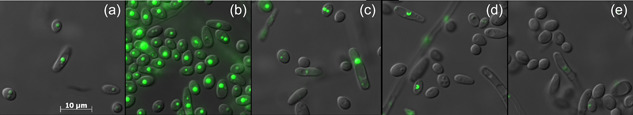
Evaluation of lipid body) formation inhibition in Y7378 Q4 + HsDGAT2 using different concentrations of PF‐06424439. (a) 0 µg/mL, (b) 6.25 µg/mL, (c) 12.5 µg/mL, (d) 25 µg/mL, (e) 50 µg/mL.

**Figure 3:**
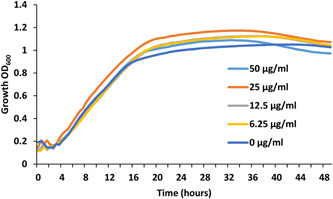
Y7378 (Q4 + HsDGAT2) representative growth curve on microtiter plate with increasing concentration of PF‐06424439.

**Figure 4:**
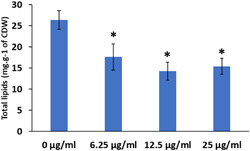
Total lipid content of Y7378 (Q4 + HsDGAT2) exposed to different concentrations of PF‐06424439 inhibitor in flask culture, evaluated by gas chromatography. Each concentration of inhibitor was evaluated against the control (0 µg/mL). Asterisks correspond to *p*‐value < 0.05.

As the HsDGAT2 is functional in *Y. lipolytica*, we therefore evaluated FP‐06424439, a specific inhibitor of DGAT2, for its capacity to inhibit the LBs formation complementation phenotype in yeast. The serial concentration of FP‐06424439 was tested against the strain Y7378 overexpressing the Human DGAT2. In these experiments, we increase the C/N ratio in the medium to 30 as it increases the TAG accumulation in *Y. lipolytica* (Gajdoš et al., 2016) and will consequently improve LB visualization. Accordingly, LBs appear bigger in the Y7378 strain without treatment (Figure 2). Small inhibition of LB formation can be observed at 12.5 µg/mL and increases at 25 µg/mL, where almost no LBs can be observed. At 50 µg/mL, no LBs are formed (Figure 2). The different concentrations of PF‐06424439 have no impact on the fitness of the strain as growth is not affected for all the concentrations tested (Figure 3). Exposition to inhibitor PF‐06424439 was also performed in larger volume in a flask for total lipids quantification using gas chromatography. In this condition, a strong and significant drop in cell lipid content is triggered starting from 6.25 µg/mL of PF‐06424439 confirming the effect of the inhibitor (Figure 4). Lipid content continues to drop up to 12.5 µg/mL but not significantly compared to 6.25 µg/mL (Figure 4). The effect of the inhibitors is more effective in this culture condition.

### 3.3 Specificity of DGAT inhibitors on DGATs from different origins

**Figure 5:**
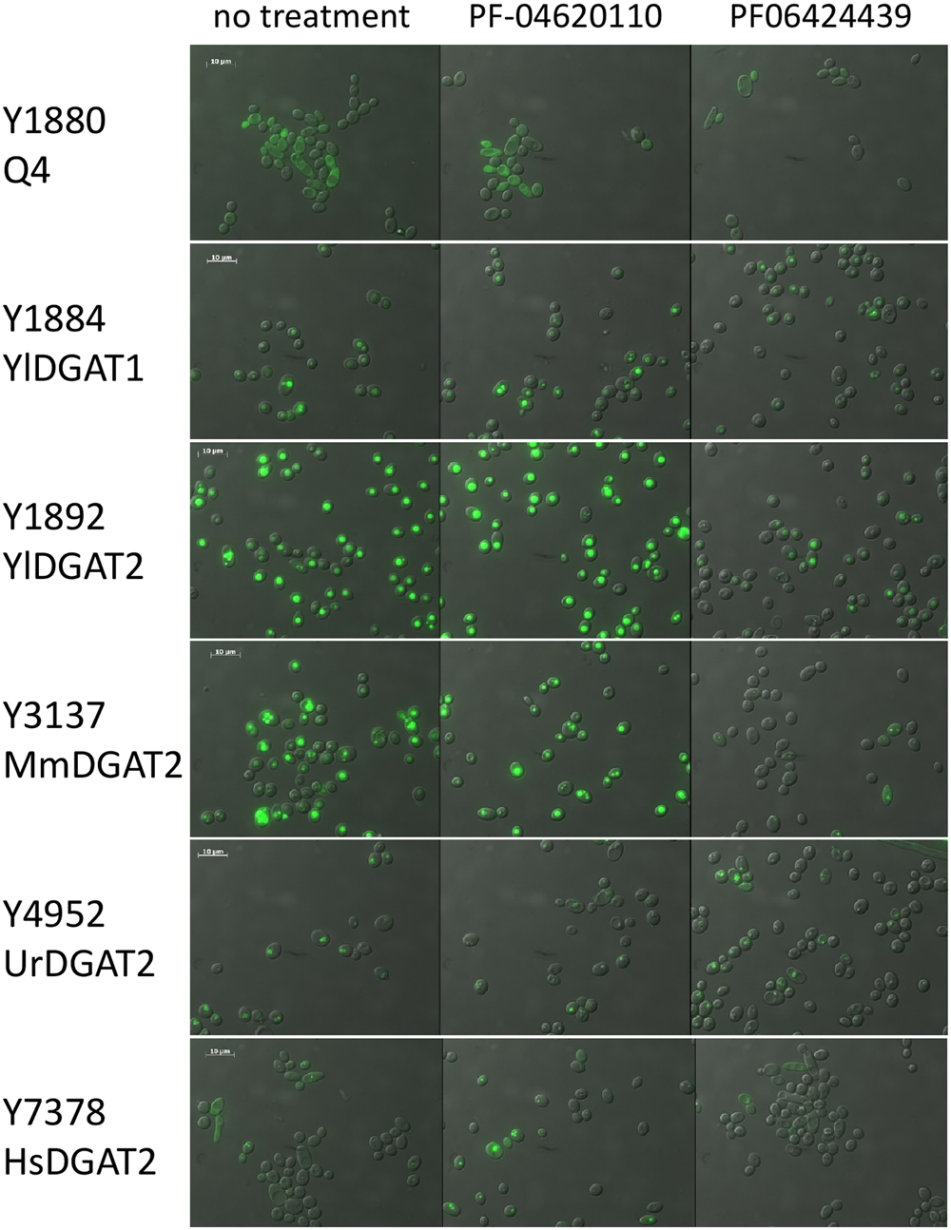
Evaluation of lipid body formation inhibition in the strains overexpressing different diacylglycerol acyltransferases (DGATs) using PF‐06424439 and PF‐046020110 as inhibitors at 25 µg/mL.

**Figure 6:**
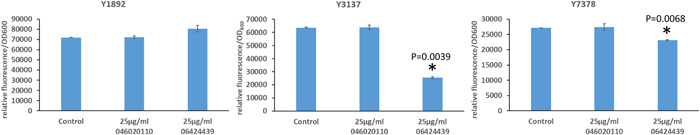
Ratio of relative fluorescence/OD_600_ of strains Y1892 (Q4 + YlDGAT2), Y3137 (Q4 + MmDGAT2), and Y7378 (Q4 + HsDGAT2) exposed to 25 µg/mL of inhibitors. Asterisks correspond to *p*‐value < 0.05.

**Figure A2:**
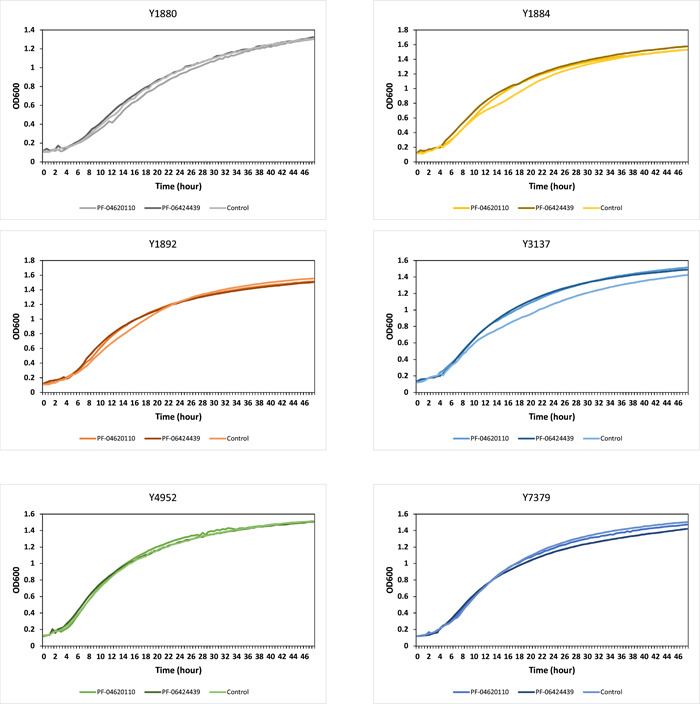
Representative growth curve on microtiter plate for the different strains exposed to inhibitors PF‐06424439 and PF‐04620110.

**Figure A3:**
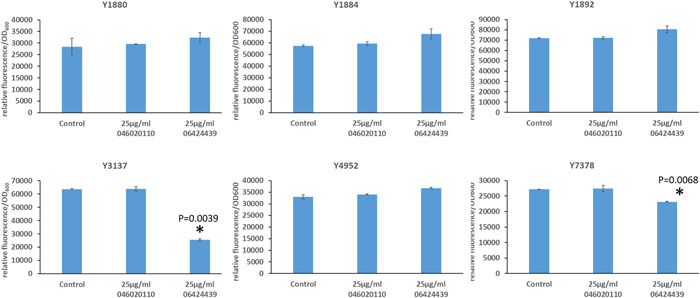
The ratio of relative fluorescence/OD_600_ of strains Y1880, Y1892, Y3137, and Y7378 exposed to 25 µg/mL of inhibitors. Asterisks correspond to *p*‐value < 0.05.

The previous experiment established a minimal concentration that inhibits LB formation without affecting growth for the DGAT2‐specific inhibitor PF‐06424439. This is also in the range of concentrations for which LBs are reduced in MCF7 breast cancer without affecting cell viability (Nisticò et al., 2021). Therefore, we tested this inhibitor at the same concentration on the six DGAT overexpressing strains selected in this study to evaluate the DGAT specificity. PF‐046020110, a DGAT1‐specific inhibitor, was also evaluated as a control. PF‐046020110 does not affect the different strains overexpressing DGAT, even YlDGAT1, at the concentration tested (Figure 5). Note, PF‐06424439 inhibits LB formation of the strain overexpressing the HsDGAT2 as demonstrated in Figure 2, and has a similar effect on MmDGAT2, while no inhibition was observed for the other strains overexpressing *Y. lipolytica* DGATs or *U. rhamaniana* DGAT2, indicating specificity for mammalian DGAT2 (Figure 5). No significant growth defects were observed for all strains with any of the compounds (Figure A2). Quantification of fluorescence was also performed in 96‐well plate cultures with a microtiter plate reader for the different strains. Results show the same trends, with a strong reduction of fluorescence observed for the strain Y3137 exposed to PF‐06424439 and a less pronounced but significant reduction for Y7378 (Figure 6). For the latter, the basal level of fluorescence is much lower, in line with fluorescent microscopy observations, which also explains the lower amplitude of reduction. For the other strains, no significant reduction was observed in agreement with fluorescent microscopy observations. (Figure A3).

## 4 CONCLUSION

Here, we provide strong arguments that *Y. lipolytica* can serve as an efficient platform for the expression and study of heterologous DGATs and the screening of therapeutic molecules targeting these enzymes. The mammalian heterologous DGATs cloned here are from cDNA libraries and are not optimized for expression in *Y. lipolytica*. Nevertheless, we show that the variants used are functional and the sequences are sufficiently conserved to retain activity in the heterologous host while keeping the drug inhibitor specificity. Thus, this strain platform is perfectly suited to screen compound libraries as mammalian DGAT2 react in the same way as in their natural environment. Modulation of the expression level of HsDGAT2 may improve the LB formation and potentially the sensitivity of this tool and will be a focus of future studies. In addition, the proof of principle presented here is validated in a microtiter plate, which is adapted to high throughput screening thanks to the simple phenotype evaluation.

## AUTHOR CONTRIBUTIONS

**Peter Gajdoš**: Investigation (equal); Writing – review & editing (equal). **Rodrigo Ledesma‐Amaro**: Investigation (equal); Writing – review & editing (equal). **Jean‐Marc Nicaud**: Conceptualization (equal); Writing – review & editing (equal). **Tristan Rossignol**: Conceptualization (equal); Investigation (equal); Writing – original draft (lead); Writing – review & editing (equal).

## CONFLICT OF INTEREST

None declared.

## ETHICS STATEMENT

None required.

## Data Availability

All data generated or analyzed during this study are included in this published article.
